# Functional Recovery of Episodic Memory After Bilateral Fornix Infarction: A Longitudinal Tractography Case Report

**DOI:** 10.7759/cureus.89708

**Published:** 2025-08-09

**Authors:** Tsukasa Koike, Sora Hamasaki, Atsumi Takenobu, Akira Teraoka

**Affiliations:** 1 Department of Neurosurgery, Teraoka Memorial Hospital, Fukuyama, JPN

**Keywords:** amnesia, anterior thalamic radiation, cingulum, fornix, frational anisotropy, fsl, mrtrix3, papez circuit, stroke, tractography

## Abstract

We present a rare case of bilateral fornix infarction in a 51-year-old woman who developed acute anterograde amnesia despite preserved general cognition. Serial neuropsychological assessments and diffusion tensor imaging-based tractography revealed a correlation between changes in fractional anisotropy (FA) and cognitive recovery, particularly in verbal and visual memory domains. Notably, Wechsler Memory Scale-Revised (WMS-R) delayed recall improved from <50 to 83, and Rey-Osterrieth complex figure test (ROCFT) delayed recall improved from 8 to 23 points during hospitalization. Tractography enabled standardized and reproducible analysis not only of the fornix but also of Papez circuit-related tracts, including the anterior thalamic radiation and cingulum subdivisions. Longitudinal reductions in FA were observed in the fornices, likely reflecting Wallerian degeneration, whereas concurrent cognitive improvement suggested functional reorganization.

This case highlights the potential of tractography-derived metrics as biomarkers of structural integrity and cognitive prognosis following focal injury to memory-critical white matter pathways. These findings may inform the development of imaging-guided strategies for monitoring and supporting cognitive rehabilitation in patients with strategic white matter lesions.

## Introduction

The fornix is a major white matter tract that connects the hippocampus to the mammillary bodies and forms a core component of the Papez circuit, which plays a central role in memory processing [1]. This anatomical structure is essential for the relay of information between medial temporal lobe structures and diencephalic memory centers. Specifically, it facilitates the transmission of hippocampal output to the mammillary bodies and subsequently to the anterior thalamic nuclei, completing a critical loop for the consolidation and retrieval of episodic memory [2,3]. Although infarction involving the fornix is rare compared to other stroke syndromes, it can lead to striking cognitive impairments, particularly severe anterograde amnesia, in the absence of motor or sensory deficits. Due to its deep midline location and fine caliber, fornix injury is often underdiagnosed or misattributed, especially in the acute phase when clinical signs are subtle [4].

Advancements in diffusion tensor imaging (DTI) and tractography have enabled more precise and noninvasive visualization of white matter architecture. Among DTI-derived metrics, fractional anisotropy (FA) serves as a sensitive indicator of microstructural integrity, reflecting changes due to demyelination, axonal loss, or edema [5]. In the context of fornix injury, reductions in FA may signal early structural compromise and, when monitored longitudinally, offer insight into dynamic neural repair or degeneration. Tractography thus not only aids anatomical localization of injury but also holds promise as a biomarker for recovery trajectories [4].

While several case reports and small case series have described fornix infarction as an etiology of isolated memory disturbance, most often manifesting as anterograde amnesia, only a limited number of studies have examined the structural-functional relationships over time [6]. In particular, the longitudinal studies that integrate serial tractographic analysis with comprehensive neuropsychological assessment remain scarce. This gap in the literature limits our understanding of the temporal trajectory of memory recovery following fornix injury, as well as the degree to which structural plasticity in white matter tracts corresponds to clinical improvement. However, previous reports of tractography focusing on the fornix have used manual techniques, making reproducibility an issue. In previous reports focusing on the fornix, regions of interest (ROIs) were often manually defined, resulting in issues with reproducibility. Furthermore, the white matter fibers analyzed were typically limited to the fornix itself, and changes in FA values across other Papez circuit-related white matter tracts were rarely addressed [5,7].

To address these limitations, we present a rare case of bilateral fornix infarction manifesting as isolated episodic memory impairment with otherwise preserved cognition. The patient underwent serial neuropsychological testing and tractography over a defined follow-up period. Importantly, we employed the XTRACT toolbox to extract and analyze multiple Papez circuit-related white matter tracts in a standardized MNI space. This approach enabled robust, reproducible quantification of tract-specific FA values beyond the fornix itself [8-10]. By correlating these objective imaging biomarkers with clinical recovery, this case highlights the feasibility and clinical relevance of using standardized tractography to monitor memory-related white matter injury.

## Case presentation

A 51-year-old right-handed woman with a known but untreated history of hypertension, previously identified during workplace health screenings, experienced acute behavioral changes while working at a confectionery shop. Her co-workers noticed that she repeatedly asked about the same tasks and appeared confused. Although she was able to speak, she complained of being unable to recall recent conversations. Observing her in a dreamlike state, her co-workers contacted emergency medical services. Upon arrival at the emergency department, her level of consciousness was scored as Japan Coma Scale 1-2. She exhibited no focal neurological deficits, such as motor weakness or sensory disturbances. Although she was disoriented to time and age, she was able to accurately state her location, birthdate, and home address. However, her short-term memory was notably impaired, as she repeatedly asked the same questions about the reason for her admission. She reported no headache, nausea, or vomiting. Her blood pressure was markedly elevated at 230/118 mmHg. Laboratory testing revealed an elevated serum uric acid level (8.7 mg/dL), but no evidence of dyslipidemia, type 2 diabetes, or thyroid dysfunction. Levels of free triiodothyronin (T3), free thyroxine (T4), and thyroid-stimulating hormone (TSH) were within normal limits (Table 1). 

**Table 1:** Laboratory test results at admission T3: Triiodothyronine, T4: Thyroxine, TSH: thyroid-stimulating hormone

Test	Result	Reference range	Unit
Low-density lipoprotein (LDL)	158	65–163	mg/dL
High-density lipoprotein (HDL)	56	48–103	mg/dL
Total cholesterol (T-chol)	227	142–248	mg/dL
Triglycerides (TG)	74	30–117	mg/dL
Uric acid (UA)	8.7	2.6–5.5	mg/dL
Hemoglobin A1c (HbA1c) National Glycohemoglobin Standardization Program (NGSP)	5.9	4.9–6.0	%
Free T3 chemiluminescent enzyme immunoassay (CLEIA)	2.54	2.10–3.10	pg/mL
Free T4 (CLEIA)	1.14	0.74–1.42	ng/dL
TSH (International Federation of Clinical Chemistry and Laboratory Medicine (IFCC), CLEIA)	0.667	0.610–4.230	µIU/mL

**Figure 1:**
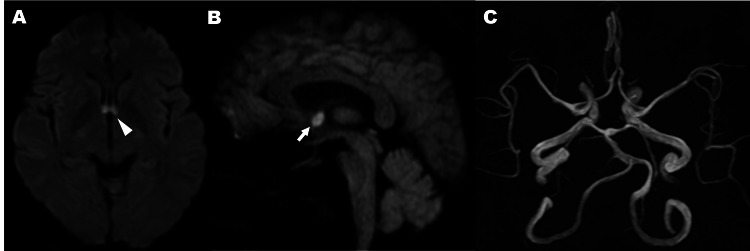
MRI findings at the time of admission A: Axial diffusion-weighted imaging (DWI) on day 0 shows bilateral fornix infarction, indicated by a white arrowhead; B: Sagittal DWI from the same day reveals that the infarct is confined to the column of the fornix (white arrow); C: Caudal view of the MRA demonstrates no stenosis or occlusion in the major cerebral arteries, particularly the anterior cerebral artery. The magnified inset in the lower right shows the anterior communicating artery. MRA: Magnetic resonance angiography

Given the patient’s markedly elevated blood pressure, absence of systemic symptoms (e.g., fever, rash, arthralgia), and no family history of thrombosis or autoimmune disease, the likelihood of vasculitis or a hypercoagulable disorder was considered low. Additionally, she had no history of medication use, including oral contraceptives, nor of smoking or alcohol consumption. Cardiac and vascular evaluations, including transthoracic echocardiography and carotid ultrasonography, revealed no valvular abnormalities, wall motion deficits, intracardiac thrombi, or evidence of unstable plaques, further reducing the suspicion of embolic or rare etiologies. Brain MRI demonstrated bilateral acute infarctions localized to the fornix, without evidence of hemorrhage. Magnetic resonance angiography (MRA) showed no major vessel stenosis or occlusion (Figure 1).

**Figure 2:**
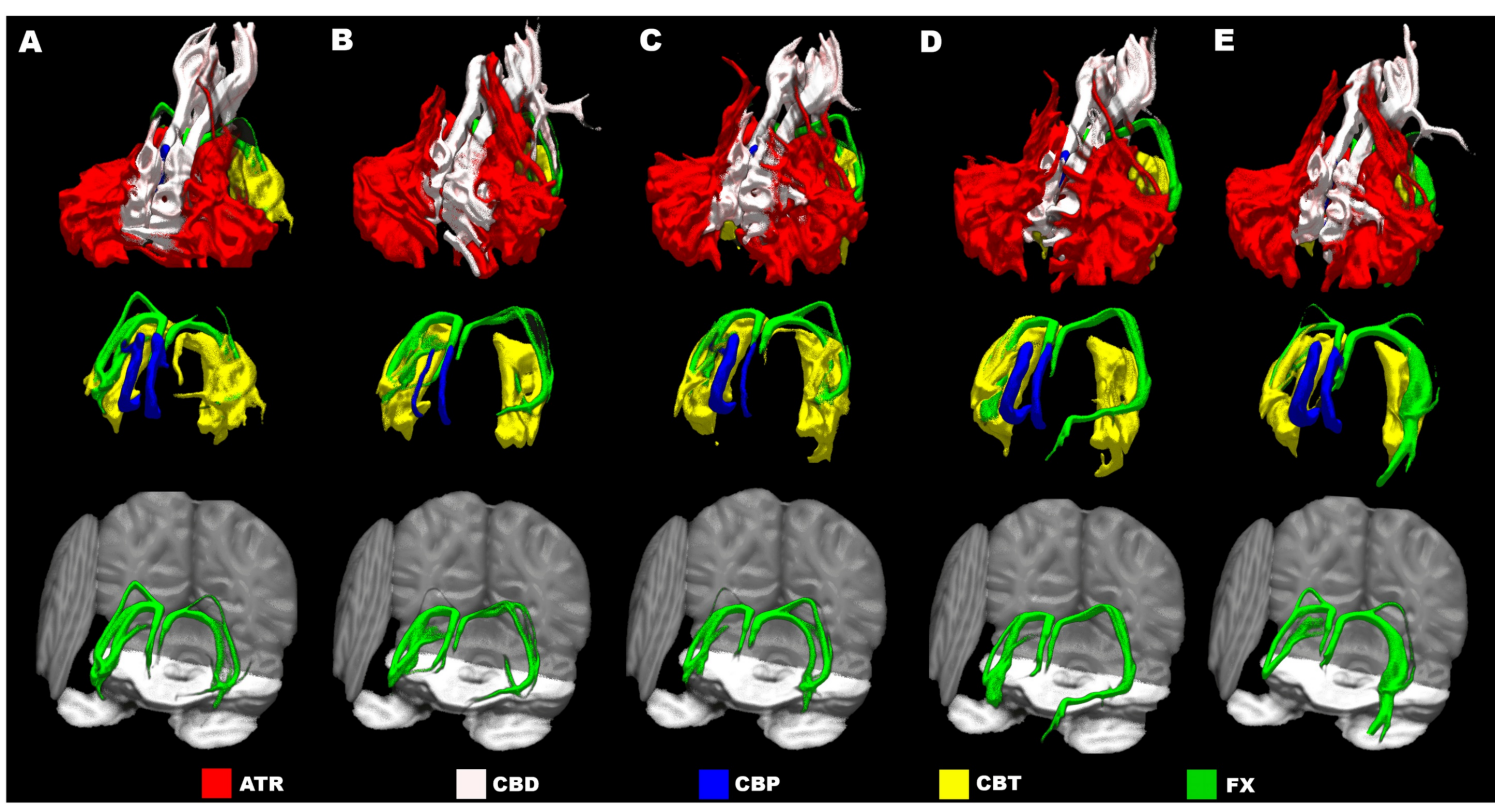
Longitudinal evaluation of tractography related to the Papez circuit Serial tractographic analysis of white matter pathways associated with the Papez circuit using XTRACT on day 1 (A), day 10 (B), day 26 (C), day 55 (D), and day 83 (E). Top row: All tracts associated with the Papez circuit; Middle row: Three tracts, namely the CBP, CPT, and FX, which are partially obscured by ATR and CBD; Bottom row: The FX tract overlaid on individual slices of T1-weighted images. Increased tract visibility, particularly in the FX (red arrowheads), corresponds to longitudinal changes in FA values. Color coding of tracts: Red for ATR, pink for CBD, blue for CBP, yellow for CBT, and green for FX FA: Fractional anisotropy, ATR: Anterior thalamic radiation, CBD: Cingulum subsection dorsal, CBP: Cingulum subsection perigenual, CBT: Cingulum subsection temporal, FX: Fornix

The findings were consistent with bilateral lacunar infarctions of the fornix. Antiplatelet therapy was initiated. No new infarctions were detected during the clinical course. In collaboration with a speech-language pathologist and a certified public psychologist, serial assessments of higher cognitive function were conducted, along with longitudinal tractographic analysis using DTI. The DTI studies were conducted at approximately two, four, and eight weeks, as well as before discharge. While the exact dates varied due to scheduling constraints, the timing was primarily based on a standardized clinical protocol for subacute stroke follow-up imaging at our institution. The FA values were monitored in the fornix, three subdivisions of the cingulum (cingulum subsection dorsal, cingulum subsection perigenual, and cingulum subsection temporal), and the anterior thalamic radiations. Longitudinal DTI-based tractography revealed decreased FA values specifically in the left fornix, with no notable changes in other major white matter tracts (Table 2, Figure 2).

**Table 2:** The transition of FA values in Papez circuit related tractography. FA: Fractional anisotropy, ATR: Anterior thalamic radiation, CBD: Cingulum subsection dorsal, CBP: Cingulum subsection perigenual, CBT: Cingulum subsection temporal, FX: Fornix, L: Left, R: Right The values are expressed as mean ± standard deviation.

FA	Day 1	Day 10	Day 26	Day 55	Day 83
ATR_L	0.36 ± 0.14	0.35 ± 0.14	0.35 ± 0.14	0.36 ± 0.14	0.34 ± 0.14
ATR_R	0.35 ± 0.13	0.34 ± 0.13	0.34 ± 0.13	0.34 ± 0.13	0.34 ± 0.14
CBD_L	0.45 ± 0.16	0.42 ± 0.16	0.42 ± 0.16	0.44 ± 0.16	0.39 ± 0.17
CBD_R	0.36 ± 0.18	0.37 ± 0.16	0.37 ± 0.16	0.36 ± 0.17	0.35 ± 0.17
CBP_L	0.38 ± 0.16	0.36 ± 0.16	0.36 ± 0.16	0.33 ± 0.15	0.40 ± 0.15
CBP_R	0.33 ± 0.15	0.36 ± 0.15	0.36 ± 0.15	0.37 ± 0.13	0.40 ± 0.16
CBT_L	0.26 ± 0.14	0.30 ± 0.13	0.30 ± 0.13	0.30 ± 0.13	0.28 ± 0.14
CBT_R	0.26 ± 0.16	0.26 ± 0.15	0.26 ± 0.15	0.26 ± 0.15	0.22 ± 0.16
FX_L	0.36 ± 0.17	0.33 ± 0.17	0.33 ± 0.17	0.34 ± 0.16	0.27 ± 0.16
FX_R	0.27 ± 0.15	0.24 ± 0.14	0.24 ± 0.14	0.21 ± 0.13	0.24 ± 0.16

Cognitive function was assessed using the mini mental state examination (MMSE), the Hasegawa dementia scale-revised (HDS-R), the trail making test-Japanese edition (TMT-J), the Rey-Osterrieth complex figure test (ROCFT), the Benton visual retention test (BVRT), and the Wechsler Memory scale-revised (WMS-R) within two weeks of admission and again within two weeks before discharge (Table 3).

**Table 3:** Transition of higher cognitive function MMSE: Mini mental state examination, HDS-R: Hasegawa dementia scale-revised, TMT-J: Trail making test-Japanese edition, ROCFT: Rey-Osterrieth complex figure test, BVRT: Benton visual retention test, WMS-R: Wechsler memory scale-revised

Test	Subtest/Measure	Hospitalization	Discharge
MMSE		25/30	30/30
HDS-R		27/30	30/30
ROCFT	Copy	36/36	36/36
	Immediate recall	11.5/36	24/36
	Delayed recall (3 minutes)	8/36	23/36
TMT-J	Part A	42	26
	Part B	88	58
BVRT	Correct responses	7	7
	Number of errors	5	3
WMS-R	Verbal memory	79	77
	Visual memory	71	112
	General memory	73	85
	Attention/concentration	100	100
	Delayed recall	<50	83

To better quantify cognitive recovery, percentage change data were calculated for key domains, including a 187.5% improvement in ROCFT delayed recall and a 57.7% increase in WMS-R visual memory. Although standard deviations for raw scores were not available, all assessments were conducted under standardized conditions. Most cognitive assessments demonstrated improvement throughout the hospitalization period, particularly in episodic memory domains such as delayed recall and visual memory. In contrast, attention-related functions, as assessed by TMT-J, remained within the borderline normal range but showed additional improvement by the time of discharge. To cope with residual short-term memory difficulties, the patient adopted compensatory strategies, such as using written notes and orientation cues. At the time of discharge, her cognitive function had recovered to a level sufficient for independent daily living, and she was discharged home in stable condition.

The MRI data acquisition, scanning protocols, and post-processing procedures were carried out. Imaging was performed using a 1.5 Tesla system (SIGNA Explorer 1.5T; GE Healthcare, Chicago, IL, USA) equipped with an eight-channel head coil. The scanning protocol comprised several sequences, including fluid-attenuated inversion recovery (FLAIR), diffusion-weighted imaging (DWI), T1-weighted imaging (T1WI), arterial spin labeling (ASL), and diffusion tensor imaging (DTI). The FLAIR sequence used a repetition time (TR) of 6.2 ms and an echo time (TE) of 0.11 ms, with a 1.0 mm slice thickness and a 512 × 512 matrix. The DWI was obtained using TR/TE of 4339/86 ms, 6 mm slices, a matrix of 256 × 256, and a b-value of 1000 s/mm². The MRA was performed with TR/TE of 25/6.8 ms, 0.8 mm slice thickness, and a matrix of 512 × 512. For T1WI, parameters included TR/TE of 14/502 ms, 1.0 mm slice thickness, and the same matrix resolution. The DTI protocol employed TR/TE of 12255/96 ms, a 2.5 mm slice thickness, and a 256 × 256 matrix, with 30 diffusion gradient directions (b = 1000 s/mm²) and one baseline image without diffusion weighting (b = 0 s/mm²). Diffusion data were processed using FSL (Functional Magnetic Resonance Imaging of the Brain (FMRIB) Analysis Group, Oxford, UK) and MRtrix3 [11,12]. Probabilistic tractography was generated, and fractional FA values were extracted using the XTRACT toolbox implemented in FSL [8,13]. This tool provides automated identification of principal white matter tracts in standardized Montreal Neurological Institute (MNI) space, facilitating cross-subject and cross-study comparisons. In addition to enhancing reproducibility, XTRACT yields quantitative indices such as tract volume, FA, and mean diffusivity, allowing for detailed characterization of microstructural integrity across specified regions of interest.

## Discussion

This case highlights the critical role of the fornix in memory encoding, consolidation, and retrieval. The patient exhibited acute and selective anterograde amnesia following a bilateral fornix infarction, a pattern consistent with disruption of the Papez circuit. Despite the absence of widespread cortical or subcortical damage, the disproportionate memory impairment underscores the fornix’s unique functional importance within the limbic memory network. Other cognitive domains, such as language, attention, and visuospatial abilities, remained relatively spared, further supporting the selective role of the fornix in episodic memory processing.

Longitudinal diffusion tensor imaging (DTI)-based tractography demonstrated a progressive decline in fractional anisotropy (FA) values in both fornices. This finding is compatible with ongoing Wallerian degeneration and secondary demyelination following the initial ischemic insult. The continued decline in FA from the subacute to chronic phases may reflect a combination of axonal loss, cytotoxic edema, and delayed microstructural disintegration affecting the limbic memory circuit.

Interestingly, despite the observed decline in FA, the patient’s neuropsychological profile revealed substantial improvement in episodic memory, especially in visual memory and delayed recall. These changes were quantitatively supported by improvements in ROCFT, WMS-R, and BVRT scores [6,14,15]. This apparent paradox of functional recovery amid structural degradation suggests the possibility of network-level compensatory mechanisms. Recruitment of alternate pathways within the extended Papez circuit or synaptic plasticity in unaffected regions may have contributed to functional restoration. The improved BVRT performance may particularly reflect compensatory adaptation in the right fornix, while gains in verbal memory could be associated with left fornix-related processes. These findings align with previous reports indicating that even in the adult brain, the limbic system retains a degree of plasticity sufficient to support cognitive recovery after focal injury [2,14].

The coexistence of declining FA values with improving memory function raises important questions regarding the interpretation of tractographic biomarkers. While FA reduction typically signals compromised microstructural integrity, functional compensation may occur via mechanisms not captured by FA alone, such as synaptic reorganization, neurochemical adaptations, or interhemispheric recruitment. Therefore, the observed trajectory suggests that functional recovery does not necessarily require complete structural restitution but may instead rely on the reorganization of preserved circuitry [15].

This case further illustrates the clinical value of longitudinal DTI-based tractography in assessing dynamic changes in white matter integrity following focal injury. Unlike conventional structural imaging, which may miss subtle or functionally critical lesions, tractography allows for region-specific, quantitative evaluation of white matter pathways. In this case, serial FA measurements served as a surrogate biomarker for functional evolution, thereby informing both prognosis and individualized rehabilitation planning. Such approaches may become increasingly important in managing complex cognitive sequelae of small vessel disease or strategically located infarctions [9,10,13].

In addition to the fornix, this case also tracked FA changes in other tracts involved in the Papez circuit, including the anterior thalamic radiation (ATR) and three subdivisions of the cingulum (dorsal, perigenual, and temporal) [16,17]. Although the ATR is not strictly confined to the Papez circuit, it encompasses thalamocingulate projections linking the anterior thalamic nuclei to the cingulate cortex, an essential component of the extended episodic memory network. Including these tracts enabled a broader view of structural plasticity and allowed for a more comprehensive assessment of the recovery process across limbic white matter pathways [17-19].

Several limitations of this study must be acknowledged. As a single-case report, the findings may not be generalizable. It is also difficult to disentangle the respective contributions of spontaneous recovery and structured rehabilitation to the observed cognitive improvements. Additionally, all imaging was performed using a 1.5 Tesla scanner, which, although widely used, offers a lower signal-to-noise ratio compared to 3.0 T systems. Nevertheless, previous studies have shown that FA values remain largely consistent across these field strengths, supporting the robustness of our observations [20].

Future investigations involving larger patient cohorts and standardized longitudinal imaging protocols are warranted to further validate the utility of tractography in predicting recovery and guiding therapeutic interventions. Tract-specific biomarkers may also aid in the early identification of patients at risk for persistent cognitive deficits and in monitoring responses to rehabilitative or pharmacologic therapies aimed at restoring white matter integrity.

## Conclusions

This case highlights how bilateral fornix infarction, although rare, can result in isolated and severe anterograde amnesia while largely sparing other cognitive domains. Serial diffusion tensor tractography offered valuable insights into both the structural disruption and evolving microstructural changes in the fornix, as reflected by longitudinal FA values. These imaging findings paralleled neuropsychological improvements over time, supporting the potential of tractography as a noninvasive tool for monitoring cognitive recovery. Although bilateral fornix infarction is uncommon, diffusion tractography may be considered in cases of unexplained acute memory disturbances, particularly when conventional imaging fails to reveal overt lesions. Moreover, this methodology may hold promise for application in other strategically located infarct syndromes or focal white matter injuries, where functional outcomes depend on damage to specific tract-based networks.
